# Oral Health Behaviors and Challenges Among Migrants in Italy: A Cross‐Sectional Study

**DOI:** 10.1155/ijod/1123956

**Published:** 2026-07-27

**Authors:** Mahdis Vakili, Carlo Gaeta, Giulia Malvicini, Simone Grandini, Tiziana Doldo

**Affiliations:** ^1^ Department of Medical Biotechnologies, University of Siena, Siena, Italy, unisi.it

**Keywords:** dental care, dental tourism, Italy, knowledge, migrant, oral hygiene, social media

## Abstract

**Background:**

Oral health disparities remain a significant public health concern among migrant populations, often driven by socioeconomic barriers, limited access to dental care, and challenges in health information acquisition.

**Objective:**

This study aimed to assess oral health behaviors, access to dental care, and sources of oral health information among migrants residing in Italy and to examine their association with the presence of untreated dental caries.

**Methods:**

A cross‐sectional study was conducted among 427 migrants living in Italy. Data were collected using a structured online questionnaire covering sociodemographic characteristics, oral health behaviors, dental care utilization, and use of social media for oral health information. The primary outcome was the presence of at least one untreated dental caries (yes/no), assessed through self‐reported data. Univariate and multivariable logistic regression analyses were performed to identify factors associated with untreated caries.

**Results:**

A total of 63.5% of participants reported having at least one untreated dental caries. Most participants reported irregular dental attendance, with a high proportion seeking care only in emergency situations. Monthly income, nationality, and frequency of dental check‐ups were significantly associated with untreated caries in univariate analysis. However, in multivariable analysis, only nationality remained significantly associated with the outcome (*p* < 0.0001). Additionally, limited use of preventive dental services and low perceived credibility of oral health information from social media were observed.

**Conclusion:**

Untreated dental caries were highly prevalent and were associated with socioeconomic and access‐related factors within the study population. These findings highlight the importance of improving access to preventive dental services and strengthening reliable oral health communication strategies in migrant populations.

## 1. Introduction

Oral diseases represent a major global health burden, affecting ~3.5 billion people worldwide, with untreated caries of permanent teeth impacting nearly 2 billion individuals. In Italy, oral health challenges persist, with a considerable prevalence of untreated caries and significant healthcare expenditure, highlighting the need for effective preventive strategies [1, 2].

Migrants are particularly vulnerable to oral health inequalities, as they often face multiple barriers to healthcare access, including language difficulties, limited insurance coverage, socioeconomic constraints, and reduced familiarity with healthcare systems. These factors, together with behavioral and social determinants, contribute to disparities in oral health outcomes within this population [3, 4]. In Italy, dental care is largely delivered through the private sector, with limited coverage by the National Health Service, leading to reduced accessibility and unmet dental needs, particularly among vulnerable groups [5–7].

The migrant population in Italy is heterogeneous, comprising individuals from diverse socioeconomic and cultural backgrounds, including labor migrants, asylum seekers, refugees, students, and individuals arriving through family reunification [2, 8, 9]. A large proportion originates from developing or less‐developed countries, where differences in economic conditions and healthcare systems may influence health‐seeking behaviors [10–12]. In this context, migrants may face complex decisions regarding dental care, including postponing treatment, seeking care within Italy, or traveling to their countries of origin. Financial constraints and structural barriers may contribute to the latter, a phenomenon commonly referred to as dental tourism [13–15].

In Italy, marked differences in oral health behaviors and access to dental care have been reported between native and migrant populations. Migrants are more likely to delay dental visits, rely on emergency care, and report unmet dental needs due to financial and structural barriers. These factors are associated with higher levels of untreated oral disease and irregular use of preventive services [6, 7, 16, 17].

Despite the growing size and diversity of the migrant population in Italy, evidence on oral health behaviors, decision‐making patterns, and sources of oral health information in this group remains limited. In particular, little is known about how socioeconomic factors and digital information channels, such as social media, influence oral health behaviors and related outcomes. Although social media is widely used as a source of health information, its effectiveness in promoting sustained behavioral change remains inconsistent [18–20].

Untreated dental caries represents a key clinical indicator of unmet dental needs in migrant populations and reflects underlying disparities in access to care and preventive practices. Understanding the interplay between socioeconomic conditions, behavioral factors, and information sources is therefore essential to address oral health inequalities in this group.

Therefore, this study aimed to assess oral health behaviors, access to dental care, and sources of oral health information among migrants in Italy and to examine their association with the presence of untreated dental caries.

## 2. Material and Methods

### 2.1. Study Design

This cross‐sectional observational study involved immigrants currently residing in Italy. Data were collected using an online questionnaire developed through Google Forms. The survey consisted of 23 questions distributed across three sections: sociodemographic characteristics, oral hygiene habits and dental care expenditure, and sources of oral health knowledge.

The data collection period extended from 17 March to 16 April 2024.

### 2.2. Participants and Eligibility Criteria

The target population included immigrants residing in Italy, regardless of gender or health status, provided that they were able to independently complete the questionnaire using a mobile phone or computer.

Inclusion criteria were defined as follows: (i) self‐reported current residence in Italy at the time of survey completion, (ii) ability to independently complete the online questionnaire, (iii) comprehension of one of the available questionnaire languages, and (iv) age ≥ 18 years. The final sample included participants aged between 18 and 78 years (mean age: 40.05 years). No minors were included in the survey.

Exclusion criteria included (i) self‐reported permanent residence outside Italy, (ii) migration to a third country without current residence in Italy, (iii) residence outside Italy for more than 6 months, (iv) loss of legal status or deportation, and (v) inability to comprehend the questionnaire languages. Individuals temporarily residing in Italy while earning income exclusively from non‐European countries (e.g., digital nomads) were also excluded.

Participants who were illiterate or visually impaired were not included, as the online survey platform did not support assisted completion.

### 2.3. Sampling Procedure and Data Collection

Participants were recruited using a nonprobabilistic convenience sampling approach through immigrant social media groups in Italy (e.g., Facebook communities of Iranians, Afghans, and other migrant populations). Group administrators were contacted, and permission was obtained before sharing the survey link.

To improve accessibility, the questionnaire was translated into seven languages (Italian, English, Arabic, Persian, Urdu, French, and Ukrainian), based on the most represented migrant groups in Italy. Translations were performed by professional translators and reviewed by native speakers.

Participants were not selected based on predefined countries of origin. The geographic distribution of respondents reflected the composition of migrant communities active within the recruitment platforms. For analytical purposes, countries of origin were grouped into broader geographic categories (Asia, Arab countries, Africa, and Ukraine) to ensure adequate subgroup size and meaningful comparisons.

Participation was voluntary and anonymous. Eligibility was assessed through self‐reported screening questions at the beginning of the questionnaire, including confirmation of residence in Italy and duration of the stay.

### 2.4. Questionnaire Development

The questionnaire was developed based on a review of the literature on migrant oral health, access to care, and health information–seeking behaviors. The draft was reviewed by three experts in dentistry and public health to ensure content validity and clarity.

Although no formal psychometric validation was performed, content validity was strengthened through expert review and multilingual adaptation.

### 2.5. Study Variables

Study variables were defined a priori based on the study objectives.

Demographic variables included age (continuous), gender (male/female), education level, employment status, immigration status, duration of residence in Italy, and possession of a national health card. Socioeconomic variables included the monthly income and annual dental care expenditure.

Oral health–related variables included toothbrushing frequency, use of dental floss and interdental brushes, smoking status, and frequency of dental check‐ups.

The primary outcome variable was the presence of at least one untreated dental carious lesion (yes/no), assessed through self‐report.

Variables related to oral health knowledge and information sources included use of social media (yes/no), preferred platform, perceived credibility of online information (five‐point Likert scale), and self‐reported implementation of information obtained from social media.

### 2.6. Statistical Analysis

Results were presented as frequencies and percentages for categorical variables and as mean ± standard deviation for continuous variables.

Univariate logistic regression analyses were performed to examine the associations between independent variables and the presence of at least one untreated carious lesion. Odds ratios (ORs) with 95% confidence intervals (CIs) were calculated. For continuous variables, ORs represent the change in odds associated with a one‐unit increase in the variable.

Variables with a *p*‐value < 0.25 in univariate analyses were included in a multivariable logistic regression model using a backward stepwise approach. Likelihood ratio tests were used to guide variable selection. A *p*‐value < 0.05 was considered statistically significant.

All analyses were performed using SPSS version 18.0.0 (SPSS Inc., Chicago, IL, USA).

### 2.7. Ethics

This study was conducted in accordance with the principles of research involving human participants and followed established standards for observational survey‐based studies commonly performed in academic clinical settings, including teaching hospitals.

Due to the anonymous and noninterventional nature of the study, and in accordance with institutional regulations, formal ethical approval and protocol registration were not required.

Participation was voluntary and anonymous. Informed consent was obtained electronically from all participants prior to questionnaire completion.

## 3. Results

Descriptive characteristics of the study population are presented in Table 1, while the results of the univariate regression analyses are shown in Table 2.

**Table 1:** Characteristics of participants (*N* = 427).

Variable	Statistics
Age; year	
Mean ± SD	40.05 ± 15.12
Gender; *n* (%)	
Male	286 (67.0)
Female	141 (33.0)
Nationality; *n* (%)	
Asia	205 (48.0)
Ukraine	48 (11.2)
Arabs	85 (19.9)
Africans	89 (20.8)
Residency duration; *n* (%)	
Less than 1 year	116 (27.2)
2–5 years	149 (34.9)
5–10 years	96 (22.5)
More than 10 years	66 (15.5)
Type of immigrant; *n* (%)	
Student	67 (15.7)
Skilled worker	202 (47.3)
Political refugee	33 (7.7)
Humanitarian immigrant	75 (17.6)
Family reunification	50 (11.7)
Having health card; *n* (%)	
Do not have	130 (30.4)
Have	297 (69.6)
Job related to health fields; *n* (%)	
No	333 (78.0)
Yes	94 (22.0)
Monthly income; *n* (%)	
Less than 500	161 (37.7)
500–1500	163 (38.2)
More than 1500	103 (24.1)
Level of education; *n* (%)	
Illiterate	85 (19.9)
High school	182 (42.6)
Bachelor	115 (26.9)
Master	32 (7.5)
Ph.D.	13 (3.0)
Having at least one untreated caries; *n* (%)	
Do not have	156 (36.5)
Have	271 (63.5)
Smoking; *n* (%)	
None	200 (46.8)
Regular	139 (32.6)
Weed	18 (4.2)
Vape	49 (11.5)
Electronic	21 (4.9)
Smoking per day; *n* (%)	
None	200 (46.8)
Less than 5	72 (16.9)
5–10	74 (17.3)
More than 10	81 (19.0)
Using dental floss; *n* (%)	
Never	99 (23.2)
Not every day	257 (60.2)
Every day	71 (16.6)
Teeth brushing; *n* (%)	
Never	12 (2.8)
Not every day	86 (20.1)
At least once	293 (68.6)
More than once	36 (8.4)
Using interdental brush; *n* (%)	
Never	239 (56.0)
Not every day	127 (29.7)
At least once	61 (14.3)
Last year money spent for dental treatments; *n* (%)	
0–10 euros	361 (84.5)
More than 10 euros	66 (15.5)
Preferred method; *n* (%)	
Public practice in Italy	101 (23.7)
Private practice in Italy	109 (25.5)
Receiving treatment in country of origin (during holidays and whenever it happens)	90 (21.1)
Receiving treatment in country of origin (traveling there with exact purpose)	69 (16.2)
Receiving dental treatment only in urgent situations	58 (13.6)
Checkup routine; *n* (%)	
Never	234 (54.8)
Every 6 months	53 (12.4)
Once a year	50 (11.7)
Every 2 years	90 (21.1)
Learning from social media; *n* (%)	
No	169 (39.6)
Yes	258 (60.4)
Platform; *n* (%)	
None	169 (39.6)
Facebook	170 (39.8)
Instagram	59 (13.8)
Twitter	3 (0.7)
TikTok	8 (1.9)
YouTube	18 (4.2)
Trustworthiness on social media; *n* (%)	
No	318 (74.5)
Maybe	59 (13.8)
Yes	50 (11.7)
Implementation from social media; *n* (%)	
No	227 (53.2)
Maybe	75 (17.6)
Yes	125 (29.3)
Credibility of information; *n* (%)	
Not at all	37 (8.7)
Not very credible	52 (12.2)
Neutral	174 (40.7)
Somewhat credible	131 (30.7)
Very credible	33 (7.7)

**Table 2:** Univariate logistic regression analysis of factors associated with having at least one untreated caries.

Variable	Having at least one untreated caries		Crude OR	CI 95% for OR	Wald statistics	*p*‐Value
	No (*n* = 156)	Yes (*n* = 271)				
Demographic						
Age; year						
Mean ± SD	40.43 ± 15.05	39.84 ± 15.19	0.99	(0.98, 1.01)	0.15	0.697
Gender; *n* (%)						
Male	104 (36)	182 (64)	Ref	—	—	—
Female	52 (37)	89 (63)	0.97	(0.67, 1.48)	0.01	0.917
Immigration characteristics						
Nationality; *n* (%)						
Asia	92 (45)	113 (55)	Ref	—	—	—
Ukraine	14 (29)	34 (71)	1.97	(1.00, 3.90)	3.85	0.05
Arabs	5 (6)	80 (94)	13.02	(5.06, 33.49)	28.37	<0.0001
Africans	45 (51)	44 (49)	0.79	(0.48, 1.31)	0.80	0.37
Residency duration; *n* (%)						
Less than 1 year	48 (41)	68 (59)	Ref	—	—	—
2–5 years	57 (38)	92 (62)	1.13	(0.69, 1.87)	0.26	0.606
5–10 years	33 (34)	63 (66)	1.34	(0.77, 2.36)	1.08	0.297
More than 10 years	18 (28)	48 (72)	1.88	(0.97, 3.62)	3.57	0.059
Type of immigrant; *n* (%)						
Student	26 (39)	41 (61)	Ref	—	—	—
Skilled worker	74 (27)	128 (73)	1.09	(0.62, 1.93)	0.10	0.750
Political refugee	11 (40)	22 (60)	1.26	(0.52, 3.04)	0.28	0.594
Humanitarian immigrant	26 (35)	49 (65)	1.19	(0.60, 2.36)	0.26	0.609
Family reunification	19 (38)	31 (62)	1.03	(0.48, 2.19)	0.00	0.929
Social characteristics						
Level of education; *n* (%)						
Illiterate	27 (32)	58 (68)	Ref	—	—	—
High school	65 (36)	117 (64)	0.83	(0.48, 1.45)	0.40	0.527
Bachelor	45 (39)	70 (61)	0.72	(0.40, 1.30)	1.14	0.284
Master	14 (44)	18 (56)	0.59	(0.26, 1.37)	1.45	0.228
Ph.D.	5 (38)	8 (62)	0.74	(0.22, 2.49)	0.22	0.632
Monthly income; *n* (%)						
Less than 500	62 (39)	99 (61)	Ref	—	—	—
500–1500	74 (45)	89 (55)	0.75	(0.48, 1.17)	1.57	0.209
More than 1500	20 (19)	83 (81)	2.59	(1.45, 4.65)	10.33	0.001
Job related to health fields; *n* (%)						
No	127 (38)	206 (62)	Ref	—	—	—
Yes	29 (31)	65 (69)	1.38	(0.84, 2.25)	1.67	0.19
Having national health card; *n* (%)						
Do not have	44 (34)	86 (66)	Ref	—	—	—
Have	112 (38)	185 (62)	0.84	(0.54, 1.30)	0.58	0.446
Annual dental expenditure; *n* (%)						
0–10 euros	136 (38)	225 (62)	Ref	—	—	—
More than 10 euros	20 (30)	46 (70)	1.39	(0.78, 2.45)	1.29	0.254
Oral health–related behaviors						
Smoking; *n* (%)						
None	71 (36)	129 (64)	Ref	—	—	—
Conventional cigarette smoker	54 (39)	85 (61)	0.86	(0.55, 1.35)	0.39	0.530
Weed	9 (50)	9 (50)	0.55	(0.20, 1.44)	1.46	0.227
Vape	12 (24)	37 (76)	1.69	(0.83, 3.46)	2.11	0.146
Electric	10 (48)	11 (52)	0.60	(0.24, 1.49)	1.84	0.277
Number of cigarettes per day; *n* (%)						
None	71 (36)	129 (64)	Ref	—	—	—
Less than 5	29 (40)	43 (60)	0.816	(0.46, 1.41)	0.51	0.471
5–10	31 (42)	43 (58)	0.763	(0.44, 1.31)	0.94	0.332
More than 10	25 (31)	56 (69)	1.23	(0.70, 2.14)	0.55	0.458
Using dental floss; *n* (%)						
Never	32 (32)	67 (68)	Ref	—	—	—
Not every day	102 (40)	155 (60)	0.72	(0.44, 1.18)	1.64	0.200
Every day	22 (31)	49 (69)	1.06	(0.55, 2.05)	0.03	0.853
Teeth brushing; *n* (%)						
Never	2 (17)	10 (83)	Ref	—	—	—
Not every day	23 (27)	63 (73)	0.54	(0.11, 2.69)	0.54	0.459
At least once	119 (41)	174 (59)	0.29	(0.06, 1.35)	2.46	0.117
More than once daily	12 (33)	24 (67)	0.40	(0.07, 2.12)	1.15	0.282
Using interdental brush; *n* (%)						
Never	79 (33)	160 (67)	Ref	—	—	—
Not every day	53 (42)	74 (58)	0.68	(0.44, 1.07)	2.69	0.101
Once daily	24 (39)	37 (61)	0.76	(0.42, 1.36)	0.85	0.357
Preferred method; *n* (%)						
Public practice in Italy	39 (39)	62 (61)	Ref	—	—	—
Private practice in Italy	37 (34)	72 (66)	1.22	(0.69, 2.15)	0.49	0.482
Receiving treatment in country of origin (during holidays and whenever it happens)	32 (36)	58 (64)	1.14	(0.63, 2.05)	0.19	0.662
Receiving treatment in country of origin (traveling there with exact purpose)	28 (41)	41 (59)	0.92	(0.49, 1.72)	0.06	0.797
Receiving dental treatment only in urgent situations	20 (34)	38 (66)	1.19	(0.60, 2.34)	0.26	0.604
Checkup routine; *n* (%)						
Never	94 (40)	140 (60)	Ref	—	—	—
Every 6 months	25 (47)	28 (53)	0.75	(0.41, 1.36)	0.86	0.351
Once a year	4 (8)	46 (92)	7.72	(2.69, 22.16)	14.43	<0.0001
Every 2 years	33 (37)	57 (63)	1.16	(0.70, 1.91)	0.33	0.563
Social media						
Platform; *n* (%)						
None	67 (40)	102 (60)	Ref	—	—	—
Facebook	57 (34)	113 (66)	1.30	(0.83, 2.02)	1.36	0.243
Instagram	28 (47)	31 (53)	0.72	(0.40, 1.32)	1.09	0.296
Other^a^	4 (14)	25 (86)	4.10	(1.36, 12.32)	6.33	0.012
Implementation from social media; *n* (%)						
No	82 (36)	145 (64)	Ref	—	—	—
Maybe	23 (31)	52 (69)	1.27	(0.73, 2.24)	0.73	0.390
Yes	51 (41)	74 (59)	0.82	(0.52, 1.28)	0.74	0.387
Credibility of information; *n* (%)						
Not at all	12 (32)	25 (68)	Ref	—	—	—
Not very credible	17 (33)	35 (67)	0.98	(0.40, 2.43)	0.00	0.979
Neutral	54 (31)	120 (69)	1.06	(0.49, 2.28)	0.02	0.86
Somewhat credible	61 (47)	70 (53)	0.55	(0.25, 1.18)	2.30	0.12
Very credible	12 (36)	21 (64)	0.84	(0.31, 2.25)	0.12	0.730
Trust ability on social media; *n* (%)						
No	126 (40)	192 (60)	Ref	—	—	—
Maybe	16 (27)	43 (73)	1.76	(0.95, 3.26)	3.25	0.071
Yes	14 (28)	36 (72)	1.68	(0.87, 3.25)	2.43	0.119

*Note*: The univariate logistic regression model result. Crude: Unadjusted.

Abbreviations: CI, confidence interval; OR, odds ratio.

^a^Other platforms included TikTok, YouTube, and Twitter.

A total of 427 participants were included in the study, of whom 33.0% were female and 67.0% were male. Overall, 63.5% (*n* = 271) of participants reported having at least one untreated dental caries, while 36.5% (*n* = 156) reported no untreated caries. The mean age was 40.05 ± 15.12 years (male: 40.28 ± 15.42 and female: 39.60 ± 14.54). The majority of participants (42.6%) had completed high school. Detailed characteristics of the study population are presented in Table 1.

Table 2 summarizes the results of the univariate logistic regression analyses examining factors associated with the presence of untreated dental caries. In univariate analysis, nationality, monthly income, frequency of dental check‐ups, and type of social media platform were significantly associated with the outcome (*p* < 0.05).

Compared with participants from Asia, the odds of having at least one untreated dental caries were higher among participants from Ukraine (OR = 1.97) and Arab countries (OR = 13.02), while participants from Africa showed lower odds (OR = 0.79). Regarding socioeconomic factors, participants with a monthly income between 500 and 1500 euros had lower odds of untreated caries compared with those earning less than 500 euros (OR ≈ 0.75), whereas those with an income greater than 1500 euros had higher odds (OR = 2.59) (Table 2).

A multivariable logistic regression analysis was conducted to explore factors associated with the presence of untreated dental caries. Variables included in the model were nationality, income, occupation, smoking status, oral hygiene behaviors, frequency of dental check‐ups, and social media–related variables.

In the adjusted analysis, nationality remained the only variable significantly associated with untreated dental caries (*p* < 0.0001), while other variables did not retain statistical significance after adjustment. Due to the exploratory nature of the analysis and the limited contribution of additional variables, detailed model estimates are not presented, and the results should be interpreted with caution.

### 3.1. Discussion

This study provides contemporary evidence on oral health behaviors, treatment‐seeking patterns, and digital information use among migrants residing in Italy. The findings reveal substantial disparities in preventive care utilization, financial investment in oral health, smoking prevalence, and access to public dental services.

#### 3.1.1. Preventive Dental Attendance and Treatment‐Seeking Behavior

In our study, 13.6% of participants reported receiving dental treatment only in urgent situations, indicating a predominantly problem‐oriented rather than preventive approach to dental care. Furthermore, only 12.4% of participants reported attending dental check‐ups every 6 months, reflecting limited engagement in routine preventive services.

These findings suggest that preventive dental culture may be less established among migrant populations in Italy, potentially due to financial barriers, structural access limitations, or differences in prior healthcare experiences. Earlier research by Angelillo et al. [16] similarly reported high caries prevalence and unmet dental treatment needs among immigrants in Italy. Although immigration patterns have evolved since 1996, the persistence of delayed care‐seeking behaviors indicates that structural inequalities may remain relevant.

Compared with Villa et al. [17], who reported that 18.6% of Italian dental patients visited a dentist every 6 months and 17.6% sought care only in case of pain, 13.6% of participants in our study reported receiving dental treatment only in urgent situations. Additionally, while 23.5% of participants in Villa et al.’s [17] study brushed at least twice daily, only 8.4% of participants in our study reported brushing more than once per day. These comparisons suggest disparities in preventive oral health behaviors.

#### 3.1.2. Financial Barriers and Expenditure Patterns

Economic constraints were evident in the reported expenditure patterns. In our study, 84.29% of participants reported spending between 0 and 10 euros on dental and healthcare in the previous year (2023). Considering that per capita dental expenditure in Italy was reported at $317 in 2020 (WHO data) [8], this level of spending may limit access to regular preventive care or routine professional visits.

Although income was statistically associated with untreated caries in the regression analysis, the relationship does not appear to be strictly linear. The presence of untreated caries in higher‐income categories suggests that financial resources alone may not fully determine oral health outcomes in this population. Behavioral patterns, dietary habits, cultural perceptions of preventive care, and healthcare utilization practices may interact with economic factors in shaping oral health status.

Therefore, while financial limitations likely represent an important structural barrier, income should be interpreted within a broader social and behavioral context rather than as an isolated determinant of caries prevalence. These findings are consistent with broader European evidence indicating that unmet dental needs among migrants are influenced by multiple interacting socioeconomic and structural determinants [6].

#### 3.1.3. Social Media as a Source of Oral Health Information

Social media use for oral health information was common among participants; however, implementation of acquired knowledge varied significantly across groups. Among Africans, Asians, Arabs, and Ukrainians, 4.76%, 50.22%, 13%, and 20%, respectively, reported implementing information obtained from social media into their oral health behaviors.

While systematic reviews [19] suggest that digital platforms can be effective tools for oral health promotion, our findings indicate that exposure does not uniformly translate into behavioral change. Moreover, the overall credibility of oral health information on social media was reported to be very low (less than 10%), suggesting limited trust in digital sources.

The distribution of preferred platforms reflected broader Italian usage patterns [21]. For example, Facebook was most frequently used among Africans (80.16%), whereas Instagram was predominant among Arabs (46%), Asians (40.26%), and Ukrainians (75.38%). These patterns may inform targeted public health communication strategies. However, low credibility presents a dual implication: Skepticism toward unreliable information may prevent harm, yet insufficient trust in accurate professional content may reduce the effectiveness of digital health promotion.

#### 3.1.4. Access to Public Dental Services and Dental Tourism

When experiencing pain or recognizing a need for treatment, only 23.56% of participants reported that they would seek care in public facilities (ASL or hospital). In contrast, 37.16% preferred to receive treatment in their country of origin.

This pattern may reflect perceived affordability differences and long waiting times in public Italian dental services. However, seeking care abroad—particularly for complex procedures such as prosthodontics or implant therapy—may introduce risks related to continuity of care and management of complications. As discussed by Ashiti et al. [15], dental tourism may present clinical and legal challenges when complications arise.

#### 3.1.5. Length of Stay and Acculturation

Although more than 60% of participants had resided in Italy for less than 5 years, those living in Italy for more than 10 years showed a higher, although not statistically significant, prevalence of untreated caries. This suggests that the duration of residence alone does not necessarily translate into improved oral health outcomes. Acculturation is a multifactorial process involving socioeconomic integration, dietary adaptation, healthcare utilization patterns, and cultural practices. Therefore, length of stay should not be interpreted as a direct proxy for successful integration into preventive healthcare systems [20].

#### 3.1.6. Smoking Prevalence

Smoking prevalence in our study was notably high, with 53.07% of participants reporting current use of traditional or electronic cigarettes, which is substantially higher than the 18.6% prevalence of current or former smokers reported in a recent Italian study [22]. Although untreated caries was the primary outcome variable, smoking was included as an oral health–related behavioral factor. While smoking is more strongly associated with periodontal disease and oral cancer than with caries development, it reflects broader health risk behaviors that may coexist with inadequate preventive practices and irregular healthcare utilization. Therefore, the elevated prevalence observed in this population suggests additional oral health vulnerabilities beyond untreated carious lesions and supports the importance of integrating smoking cessation strategies into preventive programs targeting migrant communities.

#### 3.1.7. Gender Differences

In the present study, biological gender was not significantly associated with untreated caries. This finding contrasts with previous literature reporting higher caries prevalence among women, often attributed to hormonal, physiological, and behavioral factors. However, in this sample, the distribution of males and females across geographic groups was relatively balanced, and socioeconomic variables may have exerted a stronger influence than gender alone. It is also possible that cultural practices, dietary patterns, and healthcare utilization behaviors moderated potential gender‐based differences. Further stratified analyses in larger samples may help clarify whether gender‐specific patterns emerge within particular migrant subgroups. Cultural and gender‐related determinants were not directly assessed in this study and may partially explain the absence of statistically significant gender differences.

#### 3.1.8. Contribution to Knowledge

This study contributes to the literature in three main ways:

1. It provides contemporary, Italy‐specific data on oral health behaviors and untreated caries among migrants.
2. It integrates socioeconomic variables, migration status, and digital information sources within a single analytical framework.
3. It highlights the interaction between financial vulnerability, healthcare access, digital trust, and preventive behaviors.

By examining these dimensions simultaneously, the study offers a comprehensive perspective on oral health inequalities within a growing and heterogeneous migrant population.

### 3.2. Limitations

This study has several limitations that should be considered when interpreting the findings.

First, the cross‐sectional design precludes causal inference, and the observed associations should be interpreted as exploratory rather than indicative of causal relationships.

Second, data were based on self‐reported responses and may be subject to measurement bias, including recall bias and potential misclassification. In particular, the assessment of untreated dental caries relied on participants’ self‐perception and was not clinically validated, which may have led to the underestimation or overestimation of the true prevalence.

The sample consisted of 427 participants recruited through immigrant‐focused social media groups. While this approach enabled access to diverse migrant communities, it represents a nonprobabilistic convenience sample and may introduce selection bias. Migrants with higher digital literacy and engagement in online platforms were more likely to participate, whereas individuals with limited internet access or lower digital engagement may be underrepresented.

Although the questionnaire was available in seven widely used languages (Italian, English, Arabic, Persian, Urdu, French, and Ukrainian), migrants speaking other languages or primarily using dialectal forms may not have been fully captured.

The sample size (*N* = 427) was sufficient to allow stable regression analyses; however, it does not ensure national representativeness. Therefore, findings should be interpreted as reflective of digitally reachable migrant communities in Italy rather than the entire migrant population.

Furthermore, residual confounding cannot be excluded. Although multiple demographic and behavioral variables were included in the analysis, other relevant determinants—such as dietary habits, oral health literacy, cultural practices, and access to preventive services—were not directly assessed and may have influenced the observed associations.

Dietary habits, including the frequency and timing of refined carbohydrate intake, were not evaluated, despite their well‐established role in caries development. Future research should incorporate dietary variables to better understand the interaction between socioeconomic status, cultural practices, and oral health outcomes.

Cultural beliefs, traditional oral health practices, and gender‐related behavioral norms were not specifically assessed and may partially explain the observed patterns. Interdisciplinary approaches incorporating sociocultural perspectives may provide deeper insights into these determinants.

These limitations should be considered when interpreting the findings of this study.

#### 3.2.1. Implications for Practice and Future Research

The findings suggest the need for multilevel interventions targeting oral health disparities among migrant populations. Public health strategies may include expanding access to affordable preventive dental services within the public sector, developing culturally and linguistically tailored oral health education programs, and collaborating with trusted community organizations to improve outreach.

Given the widespread use of digital platforms, evidence‐based oral health communication strategies delivered through commonly used social media platforms may improve engagement if credibility and trust are strengthened.

Future research should consider longitudinal designs to examine behavioral changes over time, probabilistic sampling strategies to enhance representativeness, and formal validation of culturally adapted assessment tools. Qualitative studies may further explore cultural perceptions of preventive dental care and trust in digital health information.

## 4. Conclusion

This study aimed to assess oral health behaviors, access to dental care, and sources of oral health information among migrants residing in Italy, with particular attention to socioeconomic and structural determinants.

The findings demonstrate that these objectives were addressed, revealing significant disparities in preventive dental attendance, financial accessibility, smoking prevalence, and reliance on emergency‐based services.

Untreated caries were associated with economic vulnerability and limited healthcare access, while social media—although widely used—demonstrated limited credibility and inconsistent behavioral implementation. These results underscore the importance of integrated public health strategies addressing affordability, accessibility, culturally tailored communication, and digital health literacy to reduce oral health inequalities among migrant populations in Italy.

## Author Contributions

Mahdis Vakili contributed to data collection and interpretation. Tiziana Doldo and Simone Grandini contributed to data interpretation and critically revised the manuscript. Carlo Gaeta contributed to the study’s conception and design and critically revised the manuscript. Giulia Malvicini contributed to the statistical analysis of the survey.

## Acknowledgments

The authors gratefully acknowledge the support of the University of Siena (Università degli Studi di Siena). This article was published Open Access thanks to the funding provided by the University of Siena within the framework of institutional agreements with publishers.

## Funding

This study was financially supported by the University of Siena, which provided full funding for the publication of this manuscript.

## Disclosure

All authors have read and agreed to the published version of the manuscript.

## Ethics Statement

This study was based on anonymous survey guidelines, and online informed consent was obtained from all participants before the compilation of the questionnaire.

## Conflicts of Interest

The authors declare no conflicts of interest.

## Data Availability

The data that support the findings of this study are available from the corresponding author upon reasonable request.
